# Woodlands and Malaria

**Published:** 1887-03

**Authors:** Chas. Schmidt


### WOODLANDS AND MALARIA.

BY DR. CHAS. SCHMIDT.

*(Translated from the "Journal D'Hygiene.")*

The influence of woods and forests upon the augmentation or dimunition of malaria, is a subject replete with facts, which are beginning to engage the serious attention of health officers and savants.

The question remains the order of the day for the next International Health Congress, which is to be held in Vienna in 1887. It is, however, with the greatest embarrassment, that we bring to the notice of our lecturers, the resume of the Sixth Conference at the experimental Health Institute of the University of Rome, in which Prof. Tommasi-Crudel considered one of the principal sides of this important problem.

The Sixth Conference has for its subject "*The Roman Woods and Malaria.*"

There are two opinions relative to the influence of woods and forests upon malaria. The partisans of the first, mantain that forests play a most important part in generating fevers, and advocate their abolishment. The second, prevails more especially in Italy, and above all, in Rome. They hold in the Peninsula, that the woods are harmful as assisting in the development of malarial fermentation, and oppose themselves as insurmountable barriers to the dissipation of these exudations to any great distance. In Rome, however, the sentiment is in favor of the conservation and extension of forests. It is between these opposite opinions that we arrive at the truth, but certainly nearer the first than the second opinion, yet the first does not appear to be founded upon an exact and rational explanation of causes.

Doubtless, in every part of the world, we find forests very fertile of destructive fevers. Vast wooded countries, uninhabitable on account of malaria, have been made salubrious and habitable by extensive clearings. These facts are incontestable; but the explanation of it which is offered is erroneous.

Some hold that the generation of malaria results from the petrifaction of leaves, insects, and diverse organisms which in cold seasons strew the forests and become packed in beds upon the soil. But by this rule all forests should be malarious, since none are exempt from this species of decomposition which is common to all woodlands. The chilliness which overspreads most persons upon their entrance to a wood, serves, accoding to other writers, to prepare the way for the invasion of the fever. Some forests which in summer maintain an extreme freshness in their shade, are not in any manner dangerous, whilst others, growing in flat countries, badly ventilated, appear to concentrate in them all the unhealthiness of the country. Under their foliage on the other hand, in more elevated regions, this chilliness is impossible, for the temperature there is more lively than in the surrounding plains, and the forests dissipate the unhealthy airs which produce fever.

The following is the true cause of the beneficial influence of clearings in certain localities.

Forests are only indirectly conducive of ague. Of themselves they never produce malaria, they only favor its development *when they overspread a malarious region.* By intercepting the sun's rays, they interrupt the evaporation from the soil. Woodlands conserve by their superficial organic deposits coming in contact with the atmosphere, a great amount of humidity, and if they did not continually send forth feverish formentations they would be inoffensive. On the other hand, these deposites which have the property of retaining humidity, by becoming musty, favor the hatching of those noxious germs which conduce fever. As unfortunately this special infection of the soil occurs frequently in nature, the malarious woods are numerous, and numerous too the examples of acquired healthfulness by means of clearings. Once cut down, the trees no longer intercept the direct rays of the sun, the humidity in the superficial deposites diminishes, and with them the malarious fermentations. Under favorable circumstances, this fermentation may be thus completely dried up.

Whilst in old and new countries the method of clearing the forests redeems vast territories of malaria, a contrary theory is maintained in Rome, where they not only favor the preservation of forests, but also their extension.

The ignorant are apt to confound the germs of the African fever with the sirocco. The more cultivated will assure you that the malarious taints which infect us are borne upon the winds ; that forests oppose a sufficient obstacle to these poisonous currents, and that, consequently, it is needful to extend these benefical barriers, etc. * * We have demonstrated the impossibility of transporting malaria *en masse;* there is nothing of it. The mind is too much imbued with old prejudices, rooted in Italy before the days of Lancisi. It is this prejudice which it is necessary to combat.

In 1714, Michael Ange Caetani, Duke of Sermonete, wished to clear one of his forests situated in the south of Cisterne. After a good deal of opposition, the matter was referred to Pope Clement XI., to name a commission to decide it, who called to his assistance Giovanni Maria Lancisi, his physician and private chamberlain, in the character of expert. Lancisi, in two discourses, which unfortunately are still a law unto the commonalty, held that the forests were safeguards to public health. He supported this thesis by historical facts and scientific arguments. One of these is perhaps worthy of criticism.

All the temples of Esculapius, says Lancisi, the tutetary god of medicine, are environed by thick coverts and forests. In Greek and and Roman antiquity, sacred woods have always been objects of veneration. Their consecration was due to their hygienic properties, which made them safeguards against fever.

In order to prevent the destruction of the forest of Cisterne, Lancisi pretended that they served as a barrier to the Siroceo, and retained in great measure, the germs of fever with which the wind infected cities in its passage. But as Cisterne lies at an altitude of 77 metres, and the forest on the contrary was also low ground, the too ingenius expert, in order to explain its utility as a filterer, formed by the foliage of trees, planted in a very low plain, invented this superficial meteriologic doctrine.

The forest of Cisterne, which was not permitted to be cleared in 1714, was cleared thirty years ago, and its site was transformed into cultivated fields and pasturages; what was the consequence? The climate of Cisterne was ameliorated, and little by little, the country which had become almost depopulated, began to build up anew. It is not too much to say that this city has also become as salubrious as Albano and Frascati, and its healthfulness is incontestably greatly improved. This is demonstrated by the general condition of the population and the records of mortality.

Moreover, in the summer of 1879, investigation was made of these facts upon the ground and under the eyes of learned Conferencier himself, and he has published the result of these observations. The little respect which remained for the authority of Lancisi was lost in clamors, contradictions and controversies, to which a disclosure so useful gave rise, and the Minister of Agriculture, desiring to have the question settled, nominated a commission to make a careful investigation of it.

For three years the six members of this commission travelled over all the low lands bordering upon Rome. After three years of unremitting labor they presented to the minister an important and voluminous report, wherein is found, minutely set forth, verified, and unanimously concurred in, all their observations bearing upon the subject, from which we extract the following:

"The Commission has visited and studied with care all points of the Roman Province embraced in the petitions, publicntions, sanitary reports, obtainable before the commencement of the year, which affirm that the total or partial destruction of forests, woodlands, thickets, stumps of trees, etc., have a tendency to augment malaria. Not only are they unable, after the closest investigation, to report a single fact in support of these assertions, but in some instances, the facts prove just the contrary. It is certain that in no locality has the total or partial felling of forests increased or aggravated the malaria. On the contrary, in some localities, the malady has diminished by a better cultivation of the earth, and above all, by a more extensive drainage; results also obtained by the same means in localities destitute of wood.

We perceive that the clearing of forests upon mountains to the north of Argo, so harmful in the eyes of the good functionaries, in a sanitary point of view, was altogether harmless.

What has been the effect of planting the eucalyptus? Prof. Tommasi-Crudeli has never attributed to these trees the least influence upon fever. On the contrary, he says that in the southern hemisphere, where vegetation is more richly developed than in Europe, there are numberless forests of eucalyptus where, as Prof. Liversidge, of the University of Sidney, has declared, malaria obtains to a very great extent. He also mentions the futile efforts to promote health made at the instigation of the French trappers at the Three Fountains, where among vast plantations of eucalyptus, all the inmates of an agricultural penitentiary instituted in the neighborhood of the Abbey, in order to improve the sanitary state of the region, particularly the prisoners and guards were subject to fevers, more or less severe. Not an inhabitant of the Three Fountains escaped the malaria in 1882, during which, in all the rest of the Roman campagna the health was excellent and the percentage of fevers very small.

In conclusion, after our investigations, assisted by conferences with the eminent professor of the Experimental Institute of Health, of the University of Rome, it seems to me we are able to arrive at the following conclusions: All malarial districts, whether low or mountainous, should be cleared of forests.

In such districts, it would be advantageous, in certain cases, to clear the plains although they may be free from malaria.

Forests which cover the mountains are not in themselves malarious and should generally be preserved.

